# Ultra-Processed Food Consumption and Cardiometabolic Risk Factors in Children Living in Northeastern Brazil

**DOI:** 10.3390/nu16223944

**Published:** 2024-11-19

**Authors:** Cristiane Cosmo Silva-Luis, Mariana Souza Lopes, Sávio Marcelino Gomes, Palloma Karlla Cantalice Matias, Fernando Paiva Brandini, Paulo César Trindade Costa, Rúbia Cartaxo Squizato de Moraes, Vinícius José Baccin Martins, José Luiz de Brito Alves

**Affiliations:** 1 Health Sciences Center, Department of Nutrition, Campus I, Federal University of Paraíba, João Pessoa 58059-900, PB, Brazil; criscosmosilva@hotmail.com (C.C.S.-L.); marianalopes.ufpb@gmail.com (M.S.L.); savio.gomes@academico.ufpb.br (S.M.G.); pallomacm02@gmail.com (P.K.C.M.); fernandobrandininutri@gmail.com (F.P.B.); paulocesarnutricionista@gmail.com (P.C.T.C.); rubiacartaxo@gmail.com (R.C.S.d.M.); 2 Health Sciences Center, Department of Biomedicine Science, Campus I, Federal University of Paraíba, João Pessoa 58059-900, PB, Brazil; viniciusjbmartins@gmail.com

**Keywords:** industrialized foods, dyslipidemias, inflammation, alanine transaminase, childhood

## Abstract

Objective: To analyze the association between ultra-processed food (UPF) consumption and cardiometabolic, biochemical, and inflammatory risk factors in children in a metropolis in Northeast Brazil. Methods: A cross-sectional study using baseline data from a community-based controlled trial was carried out with 151 children from public schools in João Pessoa, Paraíba, Brazil aged 7 to 10 years. Dietary consumption was assessed using 24 h food recall, and UPF consumption was estimated using the NOVA classification system. Anthropometry (BMI for age), blood pressure, biochemical parameters (ALT, AST, GGT, cholesterol, LDL-c, HDL-c, triglycerides, fasting glucose, HbA1c, HOMA-IR, creatinine, urea, hs-CRP), and cytokines (IL-2, IL-4, IL-6, IL-10, IL-17a, IFN-γ, and TNF-α) were also assessed. Results: Children in the third tertile (highest UPF consumption) had higher serum concentrations of LDL-c (*p*-value = 0.04) and ALT (*p*-value = 0.01), with a trend towards higher AST (*p*-value = 0.06). Total energy (*p*-value = 0.01), trans fatty acid (*p*-value = 0.02), and sodium (*p*-value = 0.04) intakes were higher in the highest tertile, whereas protein (*p*-value < 0.01) and fiber (*p*-value < 0.01) intakes were lower. Concentrations of IL-17A (*p*-value = 0.01) and IL-10 (*p*-value = 0.04) were significantly higher in the second tertile. Multiple linear regression showed that UPF consumption was significantly associated with increased LDL-c, ALT, and AST concentrations. Conclusions: High intake of UPFs was associated with dyslipidemia, elevated liver enzymes, and inflammatory changes in children. Dietary interventions are needed to reduce UPF consumption and prevent cardiometabolic and liver disease in childhood.

## 1. Introduction

The global consumption of ultra-processed foods (UPFs) has increased significantly [1]. In countries such as the United Kingdom and the United States, about 60% of the diet consists of UPFs, while this consumption exceeds 40% in Australia and Canada, 30% in Mexico, and reaches 21.5% in Brazil population [2,3]. Especially for children and adolescents, UPF consumption accounts for between 18% and 68% of the diet in countries such as Brazil, Argentina, Chile, Colombia, Mexico, Australia, the United States and the United Kingdom [4]. These consumption rates are of concern given the strong association between UPF consumption and several adverse health outcomes, including obesity, hepatic steatosis, type 2 diabetes mellitus and metabolic syndrome [5,6], and cardiovascular disease [3,5,6,7,8,9,10,11]. These conditions are often associated with a state of low-grade inflammation that contributes to disease progression [12].

UPFs are a group of palatable foods that have undergone extensive industrial processing and have an unbalanced nutrient profile, particularly hypercaloric, rich in saturated and trans fat, sugar, and sodium, and low in essential nutrients [13,14]. With high energy density and specific orosensory properties such as soft texture and ease of chewing, these foods encourage rapid and often excessive consumption [7]. During critical developmental periods, such as childhood, consumption of UPFs can influence the nutritional quality of the diet and shape habitual preferences that tend to persist into adulthood [15]. Furthermore, UPFs often replace more nutritious and lower-calorie foods, promoting a high-calorie, nutrient-poor dietary profile that is widely recognized as a risk factor for inflammation, obesity, and chronic non-communicable diseases [7].

In addition to health effects, UPFs pose social, cultural, and environmental challenges and are considered part of a global crisis that requires urgent action [13]. Although the short and long-term health effects of UPFs have been recognized [16,17,18], there are still significant gaps regarding how the consumption of these foods affects cardiometabolic risk factors in children, especially in the context of the northeast region of Brazil. This region has specific socioeconomic characteristics and a growing prevalence of childhood obesity (5 and 10 years), ranking third among the regions of the country [19], which highlights the need for a more thorough understanding and in-depth analysis of local dietary influences. Furthermore, understanding how these risk factors manifest in childhood may contribute to early intervention strategies to promote better cardiometabolic health and reduce complications throughout life [1,20,21].

Although associations between UPF exposure and cardiometabolic outcomes are well established in adults, the evidence in children remains limited and, in some cases, controversial. Studies have shown that high UPF consumption is positively associated with higher body mass index (BMI) fasting glucose and systolic blood pressure, and lower high-density lipoprotein (HDL) cholesterol concentrations [21,22,23,24,25]; however, others have not found this association [26,27,28]. This discrepancy suggests that the association between UPF intake and cardiometabolic risk factors in children may vary according to environmental, lifestyle, and specific ethnic factors.

Given this scenario, this study aimed to assess the association between UPF consumption and cardiometabolic risk factors among children residing in Brazil’s northeastern region. To this end, the following research questions were formulated: Is UPF use associated with changes in the lipid profile of children? Is UPF use associated with elevated hepatic and inflammatory markers in children? Is high UPF use associated with adiposity indices in children? Are there differences in the nutritional profile of children’s diets according to UPF consumption? Therefore, this study aimed to test the hypothesis that high UPF consumption contributes to the early development of cardiometabolic risk factors and promotes increased LDL-c, liver enzymes, inflammatory profile and worse dietary profile in children.

## 2. Material and Methods

### 2.1. Ethical Considerations

The research followed the guidelines established in the Declaration of Helsinki and obtained approval from the Human Research Ethics Committee at the Health Sciences Center of the Federal University of Paraíba, João Pessoa, Brazil (protocol reference number 4.676.103). All procedures complied with Resolution 466/2012 of the National Health Council. This research represents a secondary analysis of a randomized trial conducted in Brazil, registered in the Brazilian Registry of Clinical Trials (ReBEC) under the identifier RBR-2mzmpxf. Parents received detailed information about the study and provided written consent for the data collection involving their children.

### 2.2. Participants and Study Design

This cross-sectional study relied on baseline data from a controlled community-based trial conducted among children from public schools in João Pessoa, Paraíba, Brazil, whose trial lasted 4 months with the provision of weekly food and nutrition education actions and physical activity practice. In 2020, a total of 23.861 students were enrolled in 97 public elementary schools across the four geographical regions (South, North, West, and East) of the João Pessoa (PB, Brazil) [29].

Initially, an internet search was conducted on the homepage of the secretary of education of João Pessoa to identify schools that covered the age group of interest for the study. Once a list of school names was compiled, the research team randomly visited the schools to present the project to the principals. Of the 20 schools contacted, 10 agreed to participate in the project. A consent form was then sent to the Municipal Department of Education of João Pessoa-PB, requesting permission to collect data. The data of this study were collected in 7 schools distributed in different regions of João Pessoa.

The research involved boys and girls aged 7 to 10 years attending public elementary schools. Inclusion criteria were age between 7 and 10 years and nutritional status according to the body mass index for age (BMI for age) assessed by the z-score of obesity (z-score > +2) and eutrophy (≥−2 z-score ≤ +1). Children were not allowed to participate in this study if they met any of the following criteria: inability to participate in anthropometric measurements, psychological or behavioral issues, use of medications, or any medical condition that could affect the analysis.

A posteriori power calculation was performed using GPower 3.1.9.7 (University of Kiel, Kiel, Germany). The calculation indicated that a sample size of 159 individuals with an effect size of 0.25 and alpha of 0.05 would provide more than 80% power, but the study had 151 individuals divided into tertiles (50, 51 and 50 individuals).

### 2.3. Data Collection

All collections were carried out at the school from March 2022 to June 2023. Trained dietitians conducted all interviews and protocols. Children and parents were informed two days in advance. They were given leaflets with information on how to prepare for the tests and exams.

### 2.4. Variables

#### 2.4.1. Ultra-Processed Food Consumption

To evaluate the UPF ingestion of the children, two 24 h food recalls (R24h) were collected on a weekday and a weekend day, with the data being tabulated and processed in the Brazil Nutri software program, version 1. During the interviews, a Global Diet photo album was used to clearly define portion sizes to minimize bias. At least a recall was self-reported with assistance from mothers or other caregivers. We computed the average energy intake (in kilocalories) over R24h. The total energy consumption of UPFs was represented as a percentage of the overall energy value of the diet, based on self-reported data from an R24 h. To evaluate the UPF consumption, tertiles of the percentage-based contribution of energy from UPF ingestion were evaluated, with the first tertile referring to the lowest consumption of UPFs and the last to the highest intake. Foods were categorized as UPFs based on the NOVA classification [30].

The criteria for including nutrients in the analyses were based on their importance to children’s health, particularly in relation to cardiometabolic risk [31]. Thus, trans fatty acids, dietary fiber, sodium and saturated fats were included because of their proven association with obesity, cardiovascular disease and inflammatory processes, conditions often exacerbated by the high consumption of ultra-processed foods. For fiber, intakes for energy, the Food and Nutrition Board (FNB), Institute of Medicine (IOM) and Dietary Reference Intake (DRI) recommendations of 14 g per 1000 kcal for all age groups were followed [31].

#### 2.4.2. Anthropometry Assessment and Body Composition

A scale (Omron^®^, HBF-514C, São Paulo, Brazil) was used to measure body weight, and height was obtained using a stadiometer (Alturaexata^®^, Belo Horizonte, Brazil). The assessment of nutritional status was based on body mass index for age (BMI for age) and sex, following World Health Organization (WHO) standards, with analysis conducted via Anthro Plus (version 1.0.4; WHO, Geneva, Switzerland). Children were classified as follows: obesity (z-score > +2) and normal weight (≥−2 z-score ≤ +1) [32].

Waist circumference (WC) was assessed with a flexible steel tape measure (Sanny^®,^ São Paulo, Brazil) ranging from 0 to 200 cm with an accuracy of 0.1 mm. Measurements were made in triplicate, and the arithmetic mean was recorded. To determine body fat percentage (BFP), skinfold thickness measurements were taken three times on the right side of the body using a scientific adipometer (Sanny^®^), also with 0.1 mm precision. Triceps and subscapular skinfolds were measured and the body fat percentage (BFP) was calculated according to Slaughter [33].

#### 2.4.3. Blood Pressure Measurement

A qualified nurse took blood samples after 12 h of fasting and no strenuous exercise in the previous 24 h. Blood pressure was assessed using a digital sphygmomanometer (Omron Healthcare^®^ HBP-1100, São Paulo, Brazil), which has been validated for use in children by the Brazilian Society of Cardiology. After 5 min of stabilization, three consecutive measurements were taken for each child at one-minute intervals, with the average values used for analysis. Measurements of systolic blood pressure (SBP), diastolic blood pressure (DBP), and heart rate (HR) were conducted while the child was seated with an appropriate cuff. Additionally, the arm was positioned at heart level, resting on a surface with the palm facing upward [34].

#### 2.4.4. Blood Samples, Biochemical and Cytokines Measurements

Serum concentrations of triglycerides, cholesterol, high-density lipoprotein cholesterol (HDL-c), low-density lipoprotein cholesterol (LDL-c), fasting glucose, glycated hemoglobin (HbA1c), alanine transaminase (ALT), aspartate aminotransferase (AST), gamma-glutamyl transferase (GGT), creatinine, urea, and high-sensitivity C-reactive protein (hs-CRP) were processed using an automated analyzer (Lab-Max 240, Labtest, Lagoa Santa, MG, Brazil) with standardized reagent kits, adhering strictly to the manufacturer’s guidelines. The homeostasis model assessment of insulin resistance (HOMA-IR) calculator from Oxford was used to evaluate insulin resistance. Cytokines were assessed employing the Cytometric Bead Array (CBA) method, as outlined in earlier research [34]. Cytokines IL-2, IL-4, IL-6, IL-10, IL-17a, IFN-γ, and TNF-α were quantified using Th1/Th2/Th17 CBA assay kits provided by Becton Dickinson Biosciences. The concentration of each cytokine (pg/mL) was determined based on the fluorescence intensity of each complex. The analysis utilized an Accuri C6 BD^®^ flow cytometer, San José, CA, USA, with CBA data processed through FCAP 1.0.1 software.

#### 2.4.5. Covariables

Sex (boy/girl), age (in years) and nutritional profile of the diet according to UPFs tertile (carbohydrates, protein, total fat, saturated fatty acids, monounsaturated fatty acids, polyunsaturated fatty acids, trans fatty acids, fiber and sodium).

### 2.5. Statistical Analysis

Data normality was assessed with the Kolmogorov–Smirnov test. Results are presented as mean (±standard deviation) or as median with (25–75 percentiles). To compare the groups based on data normality, either one-way ANOVA with Tukey’s post hoc test or Kruskal–Wallis with Dunn’s post hoc test was employed. After ANOVA analysis, statistically significant data were examined by multiple linear regression. Test models adjusted for age, sex, body mass index for age (BMI for age), body fat percentage (BFP), and saturated fatty acid intake (SFAI) were performed to predict the associations between serum concentrations of LDL, ALT, and AST, and energy-percentage-adjusted UPF consumption. Statistical analysis was conducted using GraphPad Prism^®^ (version 8.01), with significance set at *p* < 0.05. All assumptions of independent variables and outcome, normality of the errors, homogeneity of variances of the error, independence of the errors, and influence and collinearity of predictors have been met for the realization of the model.

## 3. Results

A total of 151 children aged 7 to 10 years—66 boys [44%] and 85 girls [56%]—were included in this study. The UPF percentage in the first tertile was 29.2 (95% CI: 26.7–31.2), in the second tertile was 39.8 (95% CI: 37.9–41.3) and the highest tertile of UPF consumption was 52.3 (95% CI: 49.1–53.8) (*p* < 0.001).

The characteristics of the study population across tertiles of energy-percentage-adjusted UPF consumption are shown in Table 1. Sex, age, nutritional status, BMI for age, WC, BFP, SBP, DBP, and HR were similar among tertiles (Table 1). Serum concentrations of FBG, HOMA-IR, HbA1C, GGT, creatinine, urea, cholesterol, triglycerides, HDL-c, and hs-CRP were similar among tertiles (Table 1). Children in the third tertile (highest UPF consumption) had higher serum concentrations of LDL-c (F = 3.07, *p* = 0.04), ALT (F = 4.32, *p* = 0.01) and a marginal association in AST (F = 2.78, *p* = 0.06) (Table 1).

The nutritional dietary profiles of participants by tertiles of UPF consumption are shown in Table 2. Children in the highest tertile of intake consumed more calories (406 kcal vs. 373 kcal, *p* = 0.01), trans fatty acids (0.4% vs. 0.2%, *p* = 0.02), and sodium (71.1 mg vs. 63.3 mg, *p* = 0.04) and less protein (2.1% vs. 2.5%, *p* < 0.01) and fiber (2.0% vs. 18%, *p* < 0.01) than those in the first tertile (Table 2).

**Figure 1:**
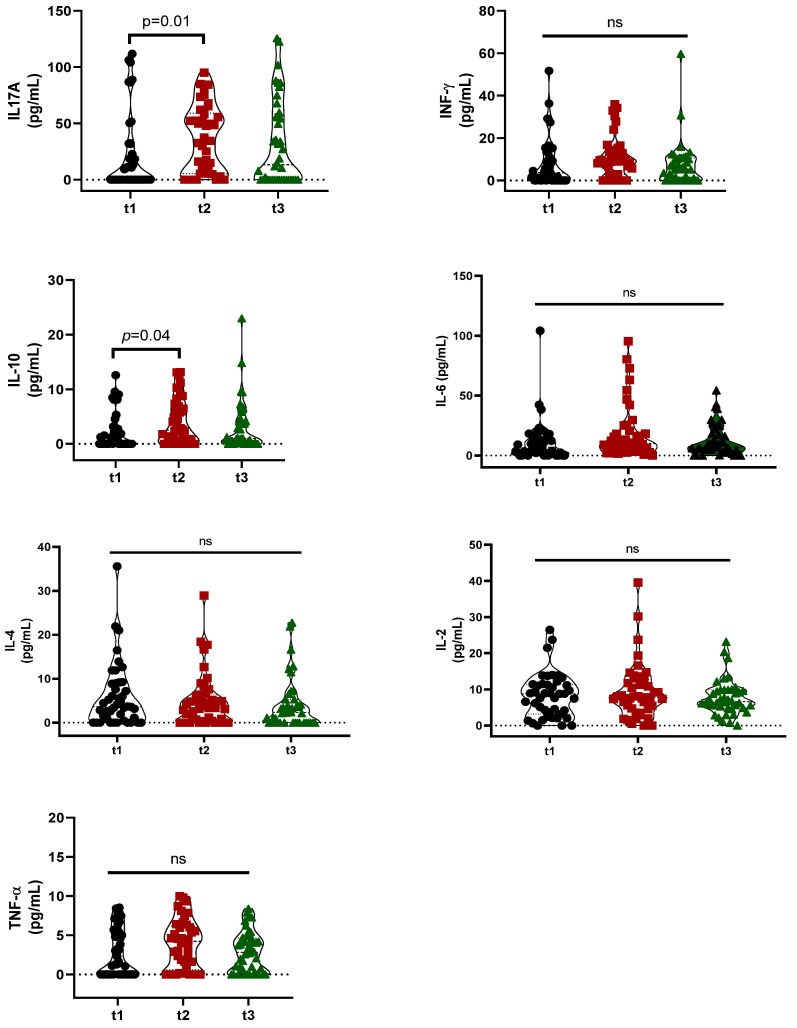
Violin plot of cytokines of participants across tertiles of energy-percentage-adjusted ultra-processed food consumption. Note: Values are expressed as median (95% CI). Kruskal–Wallis with Dunn’s post hoc was used for continuous variables. Abbreviations: Interleucine-17A (IL-17A); interferon-γ (IFN-γ); interleucine-10 (IL-10); interleucine-6 (IL-6); interleucine-4 (IL-4); interleucine-2 (IL-2); tumoral necrosis factor alpha (TNF-α); ns: no significant.

The violin plot of the cytokines IL17A, INF-γ, IL-10, IL-6, IL-4, IL-2, and TNFα across tertiles of energy-percentage-adjusted UPF consumption of 126 children are shown in Figure 1. Serum concentrations of INF-γ, IL-6, IL-4, IL-2, and TNFα were similar among groups (*p* > 0.05) (Figure 1). Children in second tertiles had higher serum concentrations of IL17A (*p* = 0.01) and IL-10 (*p* = 0.04) than those in first tertile (Figure 1).

After adjustments for age, sex, BMI for age, BFP, and SFAI remained associated with high UPF consumption (third tertile) LDL-c (β = 0.42; *p*-value = 0.01), ALT (β = 0.15; *p*-value < 0.01), and AST (β = 0.18; *p*-value < 0.05). The adjusted R^2^ for LDL-c was 0.07, for ALT 0.22 and for AST 0.12, indicating that the models explained 7%, 22% and 12% of the variation in the dependent variables, respectively, with varying levels of statistical significance (Table 3).

## 4. Discussion

The present study highlighted that the high consumption of ultra-processed foods (UPFs) among schoolchildren in the capital of Northeastern Brazil is associated with an increase in LDL-c, ALT and AST concentrations, as well as an increase in inflammatory biomarkers such as IL-17A and IL-10. We also observed an unfavorable nutritional profile in children with high UPF consumption, characterized by a high intake of trans fatty acids, low protein intake, and a dietary fiber intake of less than 14 g per 1000 kcal, which is below the minimum recommendation. These findings suggest that a high UPF diet is associated with cardiometabolic and inflammatory risk factors in children.

In Brazil, an early study showed that UPF consumption is a risk factor for high LDL concentrations in childhood [26]. In the same direction, an increase in triglycerides, lower HDL-c concentrations, and an increase in WC and fat percentage were reported in children with high UPF consumption [24,25].

In contrast, the present study found no significant changes in triglycerides, HDL-c, waist circumference, BMI for age, or fat percentage in children with high UFP consumption. These findings are in agreement with a longitudinal study performed in children aged 3–8 years in the southern region of Brazil [26]. The differences between the results may be explained by methodological and sampling differences. In addition, the amount of UPFs consumed by the children and the age groups in the different studies may also influence the metabolic responses.

ALT is a marker of hepatocellular health [35], and liver damage increases its concentrations in plasma [36]. It has been reported that AST and ALT increase with the severity of hepatic steatosis in children [37]. Metabolic dysfunction-associated steatosis liver disease is a major cause of elevated aminotransferases [3,38]. The consumption of simple carbohydrates increases ALT and liver fat [39]. Foods high in sugar and hydrogenated fats are associated with elevations in AST, ALT, triglycerides, and HOMA-IR, and increase the risk of metabolic dysfunction-associated steatosis liver disease (MASLD) [40,41].

A meta-analysis of nine studies analyzing 60.961 adults showed that moderate and high UPF consumption increased the risk of MASLD [42], due to the high intake of saturated fat and fructose [43,44,45]. Fructose presents in a large quantity of UPF is a risk factor for MASLD than glucose [46] and, together with saturated fat, predisposes children to metabolic syndrome [47]. However, in the present study, HOMA-IR, saturated fatty acids, and fasting glycemia were not significantly altered.

In our research, children with higher UPF consumption had higher dietary energy density, high concentrations of trans fatty acids and sodium, and lower protein and fiber intake. UPF consumption in children has been associated with higher energy intake and lower fiber intake [21]. Another study corroborates these findings and shows that increased UPF consumption is associated with lower fiber intake and higher energy density, factors that contribute to childhood obesity [4].

In a cohort with 4528 children at 7 years of age in the cross-sectional analysis and at 13 years of age (*n* = 3086) in the prospective analysis, UPF consumption was associated with lower protein intake and lower plasma concentrations of essential amino acids such as phenylalanine, isoleucine, leucine, and valine [25]. These findings highlight how increased consumption of UPFs can worsen diet quality, making children more susceptible to nutritional deficiencies and the risk of chronic non-communicable diseases.

It has been shown that a 100 g increase in UPF consumption increased hs-CRP by 4% in adults, independent of BMI [48]. In addition, UPF consumption was positively associated with increased serum CRP concentrations in Brazilian adolescents [49]. A previous study showed that children in the “sweet and processed” group were 44% more likely to be in the highest hs-CRP than the “healthy” group [50].

The hs-CRP is a reliable inflammatory biomarker and prognostic marker for cardiovascular events [51], and a meta-analysis of 54 prospective studies involving more than 160,000 individuals showed that elevated CRP concentrations increase the risk of heart disease and cardiovascular mortality [52]. In addition, increased consumption of ultra-processed foods (UPFs) is associated with markers of systemic inflammation, such as IL-8 [49] and IL-6 [53]. UPF consumption may promote inflammation through several mechanisms, including high intakes of sugar, salt, saturated fat, and trans-fatty acids [12].

Synthetic ingredients such as emulsifiers, thickeners, sweeteners, colors and nanoparticles, and stabilizers present in UPFs also contribute to inflammation [12,13,54]. In addition, compounds generated during processing act as pro-oxidants and pro-inflammatory [55]. The consumption of trans fatty acids and salt, as observed in our study, is particularly harmful because it stimulates inflammatory responses in macrophages and regulatory T cells [56]. High salt concentrations increase T helper 17 (TH17) cells and reduce intestinal *Lactobacillus*, which are critical for gut health and epithelial barrier integrity [56,57]. Diets high in UPFs are also associated with fat accumulation and obesity, which promote adipose tissue inflammation [58]. Eliminating UPFs can reduce systemic inflammatory markers by up to 45% [59], while diets based on minimally processed foods improve gut microbiota and immune markers [60].

### Limitations and Strengths

In terms of limitations, we sent a list to the Municipal Department of Education and Culture with the names of 10 schools that served the age group in the study, instead of requesting permission to collect data from all institutions with children in this age group, which reduces the external validity of this study.

Another issue is the potential information biases may have arisen from the use of food records, leading to an underestimation of food intake and/or alterations in typical consumption patterns during the study period. To minimize these biases, the collection was carried out over two days and photos of home measurements were used.

Despite the aforementioned limitations, our findings on the assessment of food consumption based on the degree of food processing in children living in the capital of João Pessoa, Paraíba, Northeast Brazil, reinforce the evidence that diets with high proportions of UPFs are intrinsically nutritionally unbalanced and harmful to health, being positively associated with the presence of cardiometabolic risk factors and inflammatory biomarkers in Brazilian children.

Considering the limitations of the study, some specifications for future investigations could be as follows:

1. Expanding the sample to include more schools or schools from different cities in Paraíba and other states in the northeast region, which would allow a broader representation of the association between UPF use and cardiometabolic risk factors, further increasing the robustness of the results.
2. Investigating UPF consumption in a longitudinal design, following the evolution of cardiometabolic factors over time. This type of study would allow the long-term effects of UPFs on child development and health to be observed.
3. Expanding the analysis to include other markers of inflammation and oxidative stress that may provide a more detailed view of the inflammatory and oxidative effects of UPFs on children’s bodies.
4. Exploring the impact of UPF use on children’s gut microbiota and how this impact may influence the development of cardiometabolic and inflammatory risk factors. This analysis could provide new perspectives on the pathways by which UPFs affect health.
5. Finally, considering variables related to children’s family and food environment and inequalities to understand how these influences may modify UPFS consumption and its health effects.

## 5. Conclusions

High UPF consumption is associated with elevated concentrations of LDL-c, ALT and AST, suggesting a potential risk of cardiovascular disease and liver damage in children. Exposure to UPF-rich diets may have long-lasting consequences by contributing to chronic low-grade inflammation, an important risk factor for non-communicable diseases in childhood. Therefore, it is crucial to advance research on this topic to better understand these mechanisms and to develop effective intervention strategies. This information could be useful in formulating public policies aimed at reformulating, accessing, and marketing UPFs to minimize their adverse effects and promote healthier diets from childhood.

## Figures and Tables

**Table 1:** General characteristics of study participants across tertiles of energy-percentage-adjusted ultra-processed food consumption.

		Tertiles of Energy-Percentage-Adjusted Ultra Processed Food Consumption			
Variables	All	1 (Lowest) *n* = 50	2 *n* = 51	3 (Highest) *n* = 50	*p*-Value
Sex-boys/girls (n) ^†^	66/85	22/28	23/28	21/29	0.95
Age (years)	9.0 (8.0–10.0)	8.7 (1.2)	8.9 (1.1)	9.0 (1.0)	0.41
Nutritional status (OB/NW)	97/54	35/15	32/19	30/20	0.55
BMI for age (z-score) ^†^	2.2 (0.3–2.8)	2.2 (0.6–2.9)	2.3 (0.04–2.8)	2.1 (0.4–2.7)	0.92
WC (cm)	71.2 (61.0–81.4)	73.0 (12.4)	70.4 (11.4)	72.3 (14.4)	0.56
BFP (%)	31.7 (10.6)	31.2 (9.1)	31.7 (11.5)	32.1 (11.0)	0.91
FBG (mg/dL)	80.9 (9.5)	81.2 (8.5)	79.6 (9.1)	81.9 (10.9)	0.46
HbA1C (%) ^†^	5.6 (5.4–9.6)	5.7 (5.4–6.0)	5.6 (5.4–5.9)	5.6 (5.4–5.8)	0.27
HOMA-IR ^†^	2.2 (1.3–3.2)	2.5 (1.1–3.4)	1.9 (1.2–3.0)	2.3 (1.4–3.2)	0.73
ALT (U/L)	19.2 (8.3)	17.8 (8.1)	17.7 (7.1)	22.0 (9.2) *^#^	0.01
AST (U/L)	34.9 (9.1)	33.3 (7.3)	34.0 (9.4)	37.3 (9.9)	0.06
GGT (U/L)	16.8 (7.4)	17.5 (7.5)	15.3 (7.7)	17.5 (6.9)	0.23
Creatinine (mg/dL)	0.56 (0.1)	0.6 (0.1)	0.6 (0.1)	0.6 (0.1)	0.75
Urea (mg/dL)	21.0 (6.6)	21.1 (6.6)	21.0 (7.1)	20.9 (6.3)	0.99
Cholesterol (mg/dL)	174.5 (29.9)	168.0 (19.4)	177.5 (37.7)	177.6 (28.8)	0.19
Triglycerides (mg/dL)	83.8 (33.3)	83.27 (34.9)	82.7 (34.3)	85.4 (30.9)	0.91
HDL-c (mg/dL)	52.5 (12.8)	53.3 (13.4)	53.1 (11.8)	51.1 (13.1)	0.65
LDL-c (mg/dL)	92.4 (19.6)	86.8 (15.9)	93.7 (22.2)	96.4 (19.2) *	0.04
hs-CRP (mg/dL) ^†^	1.1 (0.3–2.9)	1.0 (0.2–2.3)	1.2 (0.3–3.4)	1.2 (0.3–3.3)	0.30
SBP (mmHg)	104 (13)	105 (11.6)	103 (12.1)	103 (14.6)	0.40
DBP (mmHg) ^†^	60 (55–95)	61 (55–69)	61 (55–64)	60 (55–69)	0.89
HR (bpm)	93.8 (11.6)	96 (12.6)	92 (10.8)	93 (11.0)	0.10

Note: Values are expressed as mean (SD) or median (25–75 percentiles). One-way ANOVA with Tukey post hoc or Kruskal–Wallis with Dunn’s post hoc was used for continuous variables. * *p* < 0.05 vs. tertile 1; ^#^ *p* < 0.05 vs. tertile 2. ^†^ non-parametric data. Abbreviations: Obesity (OB); normal weight (NW); body mass index for age (BMI for age); waist circumference (WC); body fat percentage (BFP); fasting blood glucose (FBG); glycated hemoglobin (HbA1C); homeostatic model assessment for insulin resistance (HOMA-IR); alanine aminotransferase (ALT); aspartate aminotransferase (AST); gamma-glutamyl transferase (GGT); high-density lipoprotein cholesterol (HDL-c); low-density lipoprotein cholesterol (LDL-c); high-sensitivity C-reactive protein (hs-CRP); systolic blood pressure (SBP); diastolic blood pressure (DBP); and heart rate (HR).

**Table 2:** Dietary characteristics of participants across tertiles of energy-percentage-adjusted ultra-processed food consumption in the diet.

	Tertiles of Energy-Percentage-Adjusted Ultra-Processed Food Consumption			
Variables	1 (Lowest)	2	3 (Highest)	*p*-Value
	(*n* = 50)	(*n* = 51)	(*n* = 50)	
Total energy intake (Kcal/d)	1775 (373)	1998 (374) *	1981 (406) *	0.01
Carbohydrates (%)	53 (5.3)	53 (4.9)	55 (4.0)	0.24
Protein (%)	16 (2.5)	15 (2.3)	13 (2.1) *^#^	<0.01
Total Fat (%)	32 (3.2)	33 (3.2)	33 (3.8)	0.13
Saturated fatty acids (%)	10.7 (1.2)	10.8 (1.3)	10.9 (1.3)	0.82
Monounsaturated fatty acids (%)	10.1 (1.0)	10.0 (1.0)	9.9 (0.9)	0.84
Polyunsaturated fatty acids (%) ^†^	7.9 (7.5–9.1)	8.2 (7.2–8.8)	8.5 (7.4–9.4)	0.64
Trans fatty acids (%)	0.9 (0.2)	1.1 (0.3)	1.2 (0.4) *	0.02
Fiber (g/d)	19.8 (4.5)	19.1 (4.9)	18.1 (4.4)	0.22
≥14 g/1000 kcal, N° (%)	9 (18)	1 (2.0) *	1 (2.0) *	<0.01
Sodium (mg/d)	264.6 (63.3)	291.3 (72.2)	298.7 (71.1) *	0.04

Note: Values are expressed as mean (SD) or median (25–75 percentiles). One-way ANOVA with Tukey post hoc or Kruskal–Wallis with Dunn’s post hoc was used for continuous variables. * *p* < 0.05 vs. tertile 1; ^#^ *p* < 0.05 vs. tertile 2. ^†^ non-parametric data.

**Table 3:** Association between energy-percentage-adjusted ultra-processed food consumption and cardiometabolic risk factors.

	LDL-c			ALT			AST		
	β	*p*-Value	Adjusted R^2^ (*p*-Value)	β	*p*-Value	Adjusted R^2^ (*p*-Value)	β	*p*-Value	Adjusted R^2^ (*p*-Value)
UPFs	0.42	<0.01	0.07 (*p* = 0.01)	0.15	<0.01	0.22 (*p* < 0.01)	0.18	<0.01	0.12 (*p* = 0.05)
Age	−0.56	0.67		0.10	0.83		0.26	0.66	
Sex	1.78	0.58		1.89	0.14		1.9	0.19	
BMI for age	−0.90	0.18		0.39	0.14		−0.60	0.04	
BFP (%)	0.71	0.03		0.15	0.21		0.39	<0.01	
SFAI (%)	1.45	0.21		−0.05	0.91		−0.10	0.84	

Abbreviations: Low-density lipoprotein cholesterol (LDL-c); alanine aminotransferase (ALT); aspartate aminotransferase (AST); ultra-processed food (UPF); body mass index for age (BMI for age); body fat percentage (BFP); saturated fatty acid intake (SFAI). Multiple linear regression.

## Data Availability

The data that support the findings of this study are available from the corresponding author upon reasonable request. The data are not publicly available due to ethical reasons.
